# Learning to tie the knot: The acquisition of functional object representations by physical and observational experience

**DOI:** 10.1371/journal.pone.0185044

**Published:** 2017-10-12

**Authors:** Emily S. Cross, Antonia F. de C. Hamilton, Nichola Rice Cohen, Scott T. Grafton

**Affiliations:** 1 Wales Institute for Cognitive Neuroscience, School of Psychology, Bangor University, Bangor, Wales; 2 Institute of Cognitive Neuroscience, University College London, London, England; 3 Volen Center for Complex Systems, Brandeis University, Waltham, Massachusetts, United States of America; 4 Department of Psychological & Brain Sciences, University of California, Santa Barbara, California, United States of America; Universita degli Studi di Verona, ITALY

## Abstract

Here we examined neural substrates for physically and observationally learning to construct novel objects, and characterized brain regions associated with each kind of learning using fMRI. Each participant was assigned a training partner, and for five consecutive days practiced tying one group of knots (“tied” condition) or watched their partner tie different knots (“watched” condition) while a third set of knots remained untrained. Functional MRI was obtained prior to and immediately following the week of training while participants performed a visual knot-matching task. After training, a portion of left superior parietal lobule demonstrated a training by scan session interaction. This means this parietal region responded selectively to knots that participants had physically learned to tie in the post-training scan session but not the pre-training scan session. A conjunction analysis on the post-training scan data showed right intraparietal sulcus and right dorsal premotor cortex to respond when viewing images of knots from the tied and watched conditions compared to knots that were untrained during the post-training scan session. This suggests that these brain areas track both physical and observational learning. Together, the data provide preliminary evidence of engagement of brain regions associated with hand-object interactions when viewing objects associated with physical experience, and with observational experience without concurrent physical practice.

## Introduction

In order to complete an endless number of tasks in our daily lives, we rely on interactions with functional objects, whether as simple as a toothbrush or shoelaces, or as complex as a smart phone or remote control. One feature all these objects share is that the way in which we interact with them shapes our subsequent perception of them. Evidence from research with neurological patients [1–4], non-human primates [5–10], and neuroimaging with healthy human adults [11–15] provides evidence in support of this notion. Together, this rich literature demonstrates that perception of familiar functional objects, such as tools, engages brain regions required to use or manipulate these objects.

As social creatures, however, it is also the case that we watch other people manipulate certain objects that we rarely, if ever, interact with ourselves, such as a barista using a commercial espresso machine to make a latte or a butcher using a meat slicer to shave thin slices of prosciutto. To date, it remains unclear the extent to which it is necessary to *physically* interact with an object in order to build a functional representation of that object. In the present study, we made use of a knot-tying paradigm similar to that used by Cross and colleagues [16] to study how physically learning to tie a set of complex, previously novel knots compares to watching another person learn to tie a different set of knots. This paradigm allows us to test the extent to which action representations for novel objects can be acquired by observational compared to physical experience.

Most research into the relationship between objects’ functional properties and how they are perceived has focused on the perception (and/or manipulation) of common tools for which participants have a lifetime of experience [14, 17–21]. Such investigations provide crucial insights into the role of sensorimotor cortices in object perception, manipulation and tool-based imagery. An important limitation of such work, however, is that it is impossible to control or quantify participants’ prior experience with these common, everyday objects. A number of studies have aimed to address this limitation by implementing training paradigms with novel functional objects that enable precise quantification of action experience to assess the impact of *de novo* motor experience on object representations in the human brain [16, 22–27].

In a particularly novel paradigm developed by Weisberg and colleagues [28] and subsequently adapted by others [24, 27, 29], participants learned to manipulate novel tool-like objects across three training sessions and underwent functional MRI (fMRI) before and immediately after training. The task participants performed in the scanner involved discriminating between two photographs of objects to decide whether the photographs depicted the same object or two different objects. Several of these studies report training-induced changes brought about by physically interacting with these novel tools in the left inferior parietal lobule and premotor cortex [22, 24], a finding corroborated by work using similar training paradigms with different novel objects [16, 26]. Some of this work has also explored the impact of visual experience with novel functional objects perceived in a static state [24, 27]. However, the impact of watching *someone else* manipulate or interact with novel objects on brain and behavioural representations of those objects remains underexplored. According to the direction matching hypothesis (a simulationist account of action learning and perception), motor experience with an action might not be necessary to instil a motor representation of that action [30, 31]. In line with this hypothesis, visual experience alone of someone else performing an action or manipulating an object might be enough to form such associations [30, 32–36].

While a well-established developmental literature can offer valuable insights as to how physical and visual experience shape object representations in infancy [37, 38], it remains poorly understood how both types of experience impact adults’ representations of novel objects, and the extent to which physical experience is *necessary* to construct these representations. Neuroimaging work using action observation paradigms provides preliminary evidence that watching complex movements (such as dance sequences) learned by physical practice or via passive observation engages left inferior parietal lobule and right premotor cortex in a similar manner [39, 40]. In addition, behavioural data demonstrate that participants can learn to perform observed sequences at an intermediate level between physically practiced and untrained sequences [39, 40]. Related work reports that when participants undergoing fMRI observed a novel object being constructed, broad activation emerged within premotor cortex, pre-supplementary motor area, the cerebellum and basal ganglia [41]. These same authors also report that learning proficiency (assessed as participants’ ability to recreate the objects outside the scanner) positively correlated with the magnitude of neural signal within right intraparietal sulcus (IPS). This finding led the authors to suggest that the IPS is crucially involved in computing the visual to motor transformations necessary to reproduce an observed action sequence.

While this prior work sheds light on how observational experience impacts perception [39–41], these studies have all focused on the neural and behavioural consequences of observing complex, dynamic action sequences. Less is known about how physical experience compares to observational experience when perceiving action-associated objects. As mentioned above, Bellebaum and colleagues [24] included a condition in their tool learning study where participants visually explored a separate but similar set of novel tools to those they physically learned to manipulate. When participants performed the object-matching task on pairs of photographs of novel tools, prior visual experience with novel tools was associated with broad engagement of a frontoparietal network consistent with what would be engaged during object manipulation. However, direct comparison of perceiving tools from the physical compared to visual training conditions resulted in stronger activity in left inferior/middle frontal gyrus and left posterior inferior parietal lobule, suggesting that physical experience impacts core sensorimotor brain regions above and beyond visual experience with objects in a static state. A follow-up experiment by this group used the same stimuli and object-matching task and asked participants to either visually explore static images of novel tools or watch an experimenter manipulate the tools [29]. In this study, the dependent measure was mu rhythm modulation via electroencephalography [42]. The authors found that both visual exploration with no related action information as well as observational experience of the experimenter using the objects led to mu rhythm modulation over sensorimotor cortical regions when viewing static images of these objects. Although the modulation was stronger for objects from the action observation condition, the authors nonetheless conclude that visual experience alone with novel objects, whether in use or not, impacts sensorimotor cortical activity.

Taken together, the studies by Bellebaum et al. [24] and Rüther et al. [29] begin to build a case supporting the notion that observational experience with novel objects has the potential to shape sensorimotor representations of those objects. One issue that remains unresolved, however, concerns how physical and observational experience with novel functional objects *compares*, as no study to date has instilled action representations for novel objects via physical practice compared to dynamic observational experience among the same participants. In the present study, we address this by investigating how a motor representation of a novel object can be created by physical experience with the object with the intention to learn, or by observing another individual interacting with the object, without any explicit instruction to learn. Importantly for the purposes of the present study, all objects had the same function (knots) and only differed in the sequence of hand actions required to create them. Participants’ task while undergoing fMRI was the same simple perceptual discrimination task deciding whether two photographs depicted the same or different objects used by several previous studies [16, 22, 24, 27, 29], which enables us to directly compare the impact of physical and observational experience on task performance without being biased toward any one kind of experience. Both the function and visual familiarity of all objects was held constant. We manipulated how participants experienced the knots from the two main experimental categories: for one set of knots, they explicitly learned to tie them by physically practicing, and for another set of knots, they had the identical amount of passive visual exposure by watching a partner learn to tie them. Participants underwent identical fMRI sessions while performing the perceptual discrimination task immediately before and after the training manipulation. When performing the perceptual discrimination task in the scanner, it has been argued that participants draw upon whichever associated knowledge systems are available [21, 22]. We hypothesized that both physical and observational experience play important roles in object knowledge. As such, the degree of overlap (or divergence) between brain regions engaged when viewing knots from both training categories, as well as how well participants learn from watching their partners, should illuminate the degree to which both kinds of learning shape *de novo* action-based object representations.

## Materials and methods

### Participants

**Fig 1:**
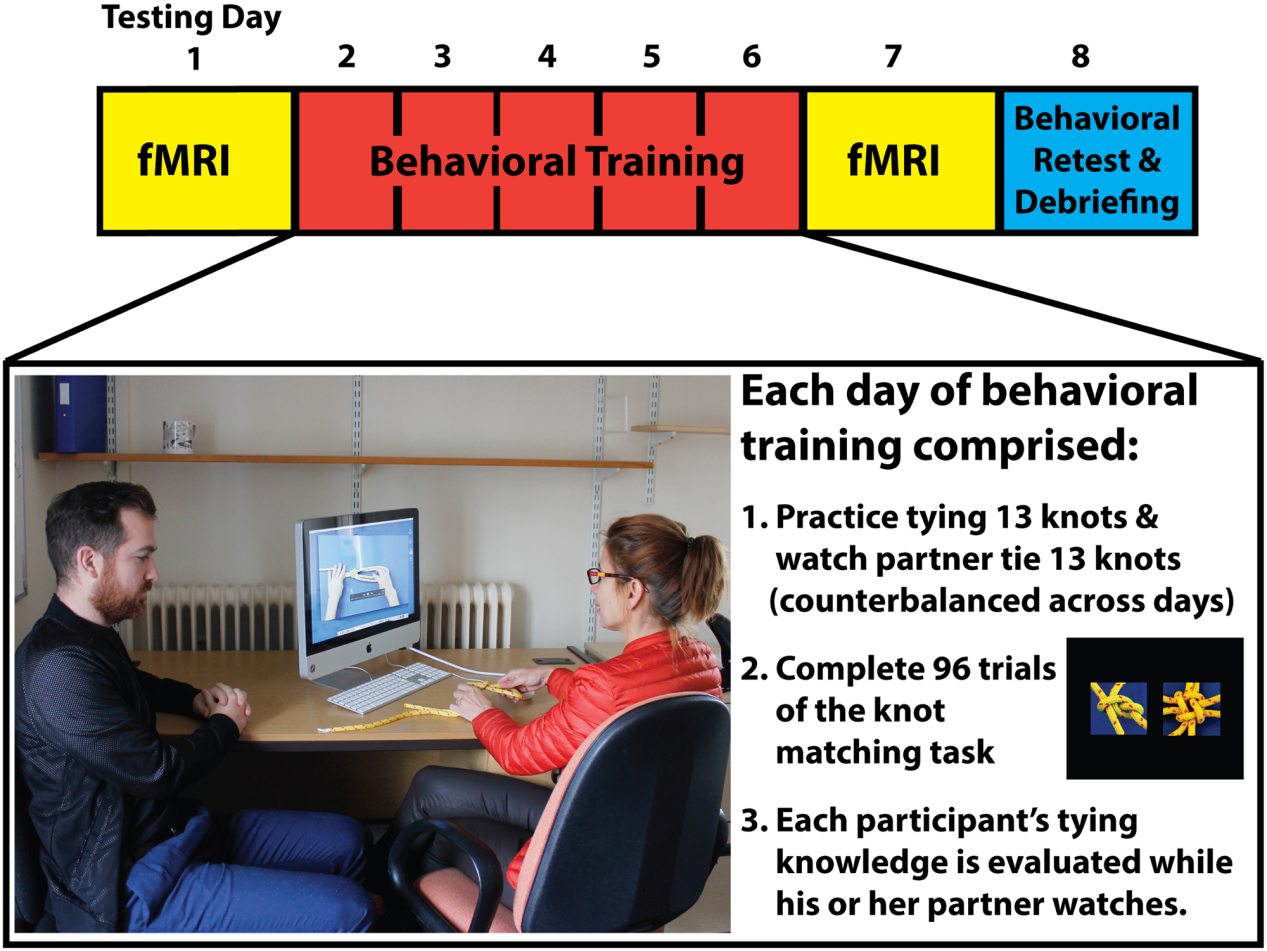
Schematic representation of the 4 phases of this study, in chronological order. All participants completed identical fMRI sessions before and after 5 consecutive days of behavioural training. Following the final fMRI session, participants returned to the laboratory for a surprise behavioural retest, where they were asked to tie the 13 knots they had physically practiced, the 13 knots they had watched their partner learn to tie, and 13 remaining knots they had never practiced or watched being tied. In the physical practice condition, in order to cater to each individual’s speed of learning, participants were able to start, stop, and restart the training videos as needed in order to recreate the 13 knots from the physical practice group. Participants were instructed to correctly tie each of these knots once per day of training, and to focus on memorizing how to tie each of these knots. When a participant observed his or her partner learning to tie their group of 13 knots, they sat next to their partner and watched their tying attempts. The computer monitor was angled in such a way that the observing partner could not see what the tying partner was watching on screen. The individuals in this photograph have provided written informed consent to publish their image.

Twenty-two undergraduate and graduate students completed all phases of the study, including behavioural training and retest procedures. Of these participants, 21 were strongly right-handed and one was left-handed, as defined by Oldfield [43]. It should be noted that we deemed the inclusion of one left-handed participant in our sample as unproblematic, due to the recent report that the majority of left-handed individuals have higher-order visuomotor functions lateralised similarly to right-handed individuals (see [44]). Participants ranged in age from 18–26 years (mean age 19.64 years), and 13 were female. Three participants were excluded from fMRI data analyses due to instabilities in the head coil, which caused signal drop out for more than 50% of one or more functional run. Of the 19 participants who composed the final fMRI sample, 18 were strongly right handed, one was left handed, and the mean age was 19.7 years (range 18–26 years). Pre-screening questionnaires ensured that all participants were neurologically healthy and had no significant prior experience with tying knots or rope work. All participants were reimbursed for taking part with cash or course credit. The Dartmouth College research ethics committee approved all procedures involved in this study (Ethical Approval Code: 20334), and all participants provided written, informed consent before taking part in any study procedures. The individuals who appear in Fig 1 have given written informed consent (as outlined in the PLoS consent form) to publish these details.

### Dyad assignment

Each participant was pseudo-randomly assigned a training partner from within the participant group. Pairs were formed based solely on availability within each partner’s schedule to come in for daily training. Once assigned, each dyad trained together across five consecutive days. Most dyads had never met each other before this experiment. Of the two dyads whose members reported having met each other previously, one dyad had taken the same class during the previous academic year, and the other dyad reported having seen each other before at the gym. Since completing this study and learning from our debriefing procedures that a number of participants thought this experiment was assessing some kind of social psychology question about watching another person learn, we realise that many fruitful questions concerning interpersonal empathy and social connection between the dyads could have been explored in this study in addition to the core questions concerning physical and visual experience with novel objects/actions. However, as we did not set out to address such questions originally, we avoid further speculation on or discussion of these issues in this paper.

### Stimuli

Stimuli comprised 39 knots that could be tied with a single piece of rope, and were a subset of those used by Cross et al. [16]. The knots were chosen based on a short pilot study where 10 knot-naïve participants (who did not participate in the present study) attempted to tie and ranked the tying difficulty of a group of 50 knots. The 39 knots that were chosen ranged in tying difficulty. To construct the instructional videos, each knot was filmed being tied by an experienced knot-tyer once at a moderate pace. The instructional videos varied in length from 5–20 seconds (depending on the complexity of the knot), and were filmed over the tyer’s hands to provide an egocentric, first-person tying perspective. Of the 39 knots, participants learned to tie 13 knots, watched their partner learn to tie 13 knots, and the remaining 13 knots remained untrained. Each group of knots was pseudo-randomly assembled to contain knots spanning a range of difficulty levels. Based on pilot data, the mean difficulty level for the knots that composed the three pseudo-randomly assembled groups did not significantly differ between the groups (all *p* values > 0.40).

In addition to the videos, high-resolution still photographs were taken of each knot from three camera angles, resulting in 117 different photographs that were used in the perceptual discrimination task for behavioural training and the fMRI portions of the study (after [16, 28]). In addition, we created scrambled images from these knot photographs that served as baseline control stimuli during scanning (c.f. [16, 24, 28]). For each knot, one phase scrambled image was generated by randomly rearranging each pixel from one of the knot images with a custom-written Matlab programme. All images were cropped and resized to 3” x 3”.

### Behavioural training procedure

The behavioural training procedures comprised three distinct tasks: explicitly learning to physically tie knots, passively watching a partner learn to tie knots, and performing the same speeded knot matching task used by Cross et al. [16]. All participants completed each of these tasks every day across five consecutive days of training, and daily training sessions lasted approximately 90 minutes.

As Fig 1 illustrates, each day of behavioural training began with participants practicing tying their 13 assigned knots and watching their partner practice his or her 13 assigned knots (Fig 1). Participants were not told to try and learn to tie their partner’s knots by observing; they were simply instructed to watch their partner complete his or her training (similar to a passive visual learning approach we employed previously; [39]. Participants had no experience with the remaining 13 knots from the group of 39. Tying and observing tasks were counterbalanced between subjects across training days. An experimenter monitored all training procedures to ensure full participant compliance during each day of training. Following the training procedures, each participant performed 96 trials of a perceptual discrimination task where they saw two photographs of knots, side by side, and had to decide whether the photographs were of the same knot or different knots. Matching knot trials always depicted the same knot from two different angles (i.e., the task could never be solved by simply template matching between the silhouettes of the two knots). These 96 trials were grouped into blocks of 16 trials from the tie, watch, and untrained conditions for each participant.

Following completion of the knot-matching task, each participant was evaluated for his or her ability to tie the knots that were practiced earlier in the session (only the knots from the ‘tie’ condition). Specifically, they were shown a static image of each knot they were required to tie, and had two attempts to tie the knot correctly. One experimenter scored all tying attempts, and assigned participants a score ranging from 0 to 5 based on their ability to tie each knot. The best score from the two attempts was taken for each knot. A score of 0 corresponded to no attempt to tie the knot, 1 corresponded to a poor, unsuccessful attempt to tie the knot, 2 corresponded to accurately performing the first steps of the knot tying correctly, 3 corresponded to half-completing the knot, 4 corresponded to almost properly tying the knot (i.e., the knot might be missing one loop or it might not be finished neatly), and a score of 5 corresponded to perfectly tying the knot in the same manner demonstrated in the training video. The other member of the dyad passively observed while his or her partner was evaluated. Order of tying evaluation between partners and across the different knots was counterbalanced across the five days of training.

After the post-training fMRI session was finished, participants returned to the laboratory and were asked to tie the group of 13 knots they had trained to tie throughout the preceding week (a measure of explicit, physical learning), as well as the 13 knots they watched their partner learn to tie (a measure of implicit, visual learning), and the 13 knots that neither they nor their partner had learned to tie (a measure of learning transfer from the physical and visual domains). Participants’ attempts to tie all of these knots were scored in an identical manner to the knots they had physically practiced tying throughout the week. Order of evaluation was counterbalanced across participants.

### Behavioural training analyses

Participants’ ability to tie the 13 knots they had practiced was evaluated by scoring their best tying attempt, averaging their tying scores for each day, and then combining across the group to create group averages. Tying scores across the five days of training were submitted to a repeated-measures ANOVA with training day as the within-subjects factor with five levels (days 1–5). Participants’ scores for tying the knots from all three training conditions (tied, watched and untrained) from the retest were submitted to a repeated-measures ANOVA to test for differences in tying aptitude across training experience.

For the knot-matching task, perceptual discrimination of the pairs of knots was assessed via mean response time and accuracy from the five days of training, and via response times while undergoing fMRI. Due to a miscoding of the button box inputs participants used to make their responses, accuracy data could not be calculated from responses made during the fMRI sessions. However, based on a previous study run by our group that used the identical knot-matching task in the scanner, we have no *a priori* reason to believe that accuracy data collected during scanning should deviate substantially from the pattern of accuracy data collected during the five days of training (see Cross et al., 2012). Response time analyses for the five days of training were restricted to only correct responses, and of these usable trials, the fastest and slowest 10% of trials were eliminated from further analyses for each testing session of 96 trials. All response time data from the scanning sessions were submitted to the outlier trimming procedure, as accuracy data were not available from the fMRI sessions. This statistical trimming procedure was done to eliminate extreme latencies in a systematic, unbiased manner (see also [16]; it should be noted that multiple approaches exist within the statistics literature for handling outliers. Opting to remove the 10% fastest and 10% slowest times is a recommended and frequently used approach to remove extreme response times while retaining the bulk of each participant’s data; see [45, 46]. In analyses where the assumption of sphericity was violated, the Greenhouse-Geisser correction was applied to the F ratio to reduce the Type 1 error rate.

### Functional magnetic resonance imaging

Each participant completed one magnetic resonance imaging session prior to the training procedures, and an identical scanning session within 48 hours of completing the five days of training (Fig 1). Participants completed three functional runs each day of scanning. The total duration of each functional run was 6 minutes and 40 seconds. Each functional run comprised 128 trials of the knot (or scrambled image) matching task, broken into blocks of 16 consecutive trials from each training condition: (to be) tied, (to be) watched, (to remain) untrained, and scrambled knot images. Seventy-five percent of trials were of knot pairs and the remaining 25% of trials were of scrambled image pairs. The stimulus blocks and matching/mismatching trials were arranged in a pseudo-random order. Within each block, half of the trials were of matching pairs of images, and half were of non-matching pairs. In each trial, a pair of knots or scrambled images appeared side by side for 2500 ms, followed by a 500 ms interstimulus interval, during which a white fixation cross appeared at a central location on the screen. As with the behavioural version of the task, participants were instructed to respond as quickly and accurately as possible once the images appeared, and their response time was recorded from the moment the stimulus appeared on the screen until a response was made.

Participants’ task during scanning was to make the same perceptual discrimination judgments they performed each day of training. Participants pressed a button held in one hand if the two knots matched, and pressed a button held in the other hand if the two photographs depicted different knots (response button assignment was counterbalanced across participants and scanning sessions). Knot-matching trials were grouped into separate blocks based on how they would be (or were) trained during the five days of behavioural training. For example, participants would perform a block of 16 trials for knots they would learn to tie, then a block of 16 trials for knots they would watch their partner learn to tie, and so on. The same grouping procedures were used for the post-training fMRI session. Debriefing procedures following the second fMRI session revealed that participants were not aware that trials had been grouped according to training condition. Interspersed within blocks of knot matching trials were blocks of trials using the phase-scrambled versions of the knot photographs (illustrations of the phase-scrambled images can be found in [16]). Participants were instructed to make the same perceptual discrimination decision during these trials. This was selected as a baseline task, as all other task parameters were held constant including attentional demands, behavioural instructions and the mean luminance of the pairs of images. It should be noted that the 3D mental rotation task participants perform with the knot photographs is different to the 2D matching task performed with the scrambled knot photographs in the baseline condition. If we were to focus on differences in brain activation when participants performed the matching task in the scanner with knot photographs compared to the scrambled baseline images, past work suggests we might see pronounced hemispheric asymmetries that would likely be less due to our training manipulation than to differences in the mental rotation task with the different stimuli (c.f., [47]), However, as the contrasts of interest in the current study focus on brain engagement when viewing and matching images of 3D knots from the different training conditions, this important difference in the visual properties between the experimental and control stimuli is less problematic.

Stimuli presentation and response recording was done by a Dell Inspiron laptop computer running Matlab Cogent 2000 Software. Stimuli were rear-projected onto a translucent screen viewed via a mirror mounted on the head coil. The experiment was carried out in a 3T Phillips Intera Achieva scanner using an eight channel phased array coil and 30 slices per TR (3.5 mm thickness, 0.5 mm gap); TR = 1988 ms; TE = 35 ms; flip angle = 90°; field of view = 24 cm; matrix = 80 x 80. For each of the three functional runs, the first two brain volumes were discarded, and the following 200 volumes were collected and stored.

### fMRI data processing and statistical analyses

Neuroimaging data from each week of scanning were first analyzed separately. Data were first realigned, unwarped and normalized to the Montreal Neurological Institute (MNI) template with a resolution of 3 x 3 x 3 mm using SPM2. A design matrix was fitted for each subject with the knots from each of the three training conditions (tied, watched, and untrained), as well as the scrambled image pairs, modelled as blocks. At the first level of fMRI analyses, single-subject fMRI responses were modelled using a standard hemodynamic response function (HRF) and its temporal derivative and dispersion derivative, aligned to the onset of a block of trials belonging to one experimental condition (i.e., tied, watched, untrained or scrambled). The design matrix weighted each raw image according to its overall variability to reduce the impact of movement artefacts [48]. After estimation, a 9 mm smoothing kernel was applied to the beta images.

The group level neuroimaging analyses were evaluated in SPM12, within a custom-made mask of sensorimotor cortices implicated in action observation and action execution [39, 49]. This mask was selected as an unbiased yet targeted way to constrain our search volume to frontoparietal regions. Group level analyses were evaluated at the p < 0.005, k = 10 voxels threshold [50]. As no results survived cluster-corrected thresholds for multiple comparisons, all results should be considered preliminary at this stage [51]. Group-level analyses were designed to achieve three main objectives:

1. The first experimental questions concerned the impact of physical practice or observational experience on viewing knots that were associated with each of these training categories compared to untrained knots. To address these questions, we performed two training experience x scanning session interactions (one for tying experience and one for observational experience). Each analysis was additionally masked by the contrast from the post-training scan session (tied > untrained) OR (watched > untrained), to isolate brain regions showing a basic training effect AND a training by session interaction (or, put another way, to reveal brain regions that are more responsive to trained compared to untrained knots after but not before training; see Weisberg et al., 2007 and Bellebaum et al., 2013 for more details on this approach). To further illustrate the nature of the BOLD response during pre-training and post-training scans, beta estimates were extracted from a sphere with a 10mm radius centred on the peak voxel from the tied and untrained conditions (for the contrast evaluating the impact of physical practice) and the watched and untrained conditions (for the contrast evaluating the impact of observational experience; see also Cross et al., 2009; 2012).
2. To investigate areas of overlap when performing the knot-matching task for the ‘tied’ and ‘watched’ conditions, a region of interest (ROI) analysis was performed on the regions that emerged from a conjunction analysis of the ‘tied’ and ‘watched’ conditions from the post-training scan session (tied > untrained ⋂ watched > untrained). This conjunction analysis selected regions where the tied > untrained and watched > untrained contrasts demonstrated regions of overlap at the *p*_*uncorrected*_ < 0.005 and *k* > 10 voxels threshold. Beta-estimates were then extracted from a sphere with a radius of 10 mm centred on the peak voxel from the overlap between tied and watched knots during the post-training scan session for all three training conditions (plus the scrambled knots baseline) and for both the pre-training and post-training scan sessions. These beta-estimates were then evaluated with a repeated-measures ANOVA to investigate differences between the individual training conditions and how they differed before and after the training manipulation. Note that this approach can result in issues with circularity. The post-training analyses will tend to find areas where the variability of the Tie and Watch conditions are low, as this is a feature of how SPM calculates t-tests. When parameter estimates are later evaluated from both the pre- and post-training scan sessions, the particularly low variability in the post-training conditions means that there is a greater chance of a significant result emerging.
3. Finally, to evaluate patterns of neural activity distinct to physical compared to observational experience when observing knots, we directly contrasted brain activity in both conditions by evaluating the contrasts ‘tied > watched’ and ‘watched > tied’, as training x scan session interactions (i.e., (Post-Training_TIED_ > Post-Training_WATCHED_) > Pre-Training_TIED_ > Pre-Training_WATCHED_) and (Post-Training_WATCHED_ > Post-Training_TIED_) > Pre-Training_WATCHED_ > Pre-Training_TIED_)).

For visualisation purposes, the sensorimotor mask is displayed on inflated cortical surfaces using the PALS dataset and Caret visualisation tools (http://brainvis.wustl.edu/wiki/index.php/Caret:About). To most clearly visualise activations from the interaction and conjunction analyses, t-images are visualised on a mean high-resolution structural scan, created from participants’ anatomical scans. Anatomical localisation of all activations was assigned based on consultation of the Anatomy Toolbox in SPM v. 2.2 [52–54] in combination with the SumsDB online search tool (http://sumsdb.wustl.edu/sums/).

## Results

### Behavioural training results

**Fig 2:**
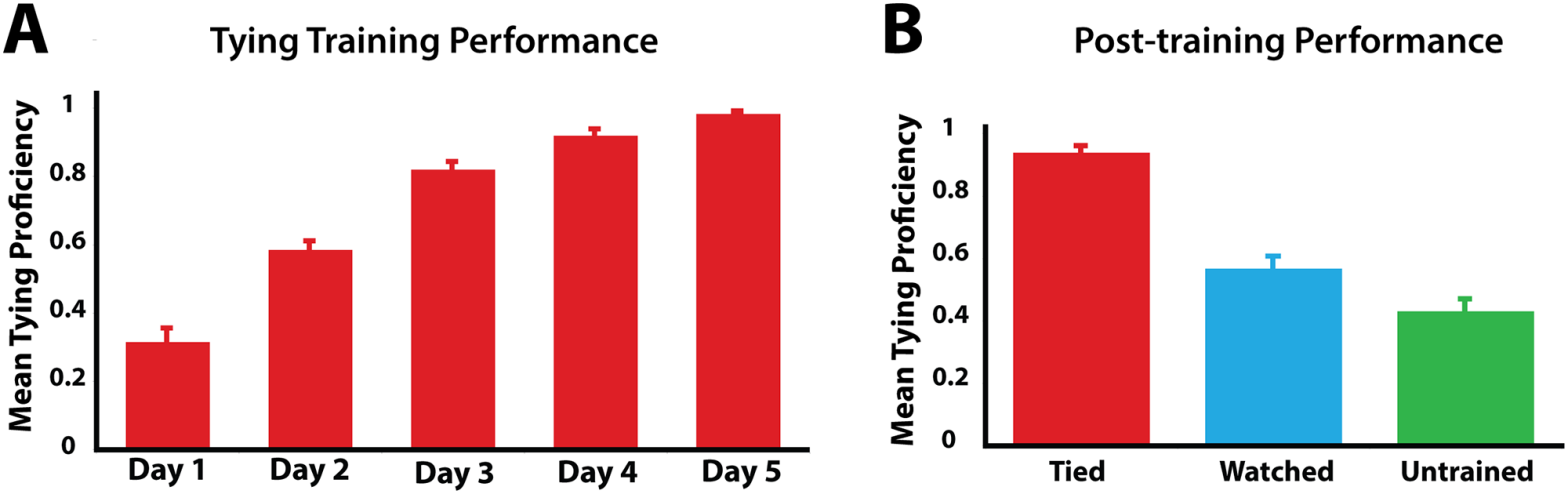
Results from the knot-training procedures. A: participants’ tying proficiency across training days. B: participants’ tying proficiency with knots from all 3 training categories during the post-training knot tying evaluation. In each plot, error bars represent the standard error of the mean.

Fig 2A illustrates participants’ performance on learning to tie the group of knots they physically practiced across five consecutive days of training. A main effect of training day is present, *F*_2.2,39.2_ = 159.52, *p* < 0.001, η^2^_p_ = 0.899, reflecting that participants’ tying performance significantly improved across time. A within-subjects contrast revealed this pattern to be a linear increase across training days, *F*_1,18_ = 314.65, *p* < 0.001, η^2^_p_ = 0.946.

### Behavioural retest results

During the post-training/post-fMRI knot tying retest, participants’ ability to tie knots from all three conditions (‘tied’, ‘watched’ and ‘untrained’) was evaluated. Results from a repeated-measures ANOVA on the three training conditions demonstrated a main effect of prior training experience on performance, *F*_2,36_ = 75.64, *p* < 0.001, η^2^_p_ = 0.808 (Fig 2B). This is best captured by a linear contrast, which demonstrates a pattern of monotonic descent based on experience, such that participants tied the knots best that they had physically trained to tie throughout the week, followed by the knots they watched their partners tie throughout the week, and they tied the untrained knots with the least proficiency, *F*_1,18_ = 122.51, *p* < 0.001, η^2^_p_ = 0.872. Pair-wise comparisons revealed that participants were significantly better at tying the knots they had physically practiced tying across the week compared to those they watched their partner tie (*p* < 0.001), or the untrained knots (*p* < 0.001). In addition, participants were significantly better at tying the knots they watched their partner learn to tie than the untrained knots (*p* = 0.002).

### Perceptual discrimination results

**Fig 3:**
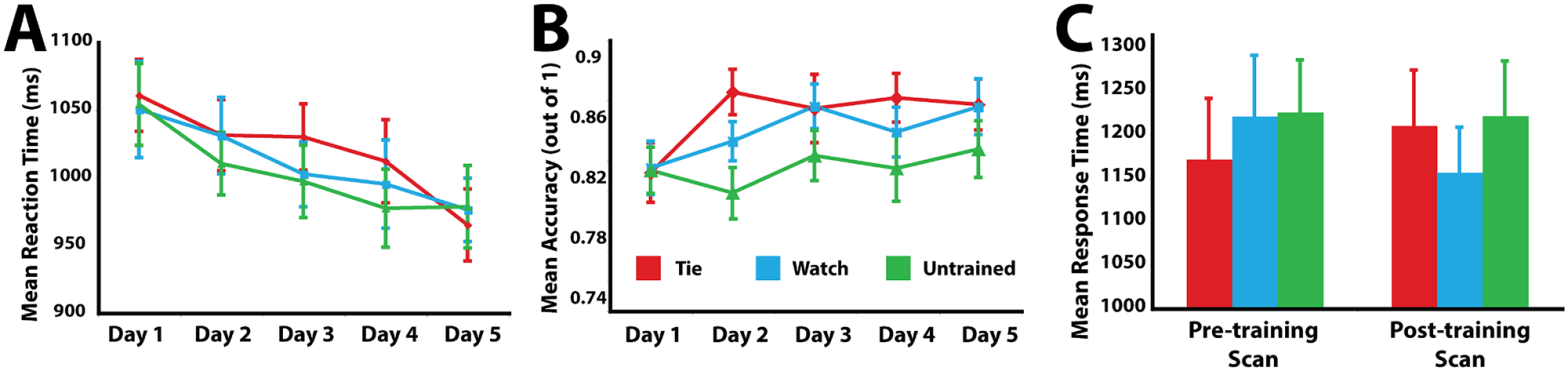
Response time and accuracy results from the perceptual discrimination (knot-matching) task. A: participants’ response time performance on the perceptual discrimination task across the five days of behavioural training. B: participants’ mean accuracy across the five days of behavioural training. C: participants’ mean response times while performing the perceptual discrimination task during the pre-training and post-training scans. In each plot, error bars represent standard error of the mean.

During the five days of behavioural training, and also during the pre- and post-training fMRI sessions, participants performed a perceptual discrimination task that required them to decide whether two photographs were of the same knot or different knots (Fig 1). The five (training day) by three (training condition) repeated-measures ANOVA performed on response times revealed a main effect of day, demonstrating that participants generally performed the task faster as the days progressed, *F*_4,72_ = 3.91, *p* = 0.006 (Fig 3A). No main effect of training was observed, nor did an interaction emerge between training and day (all *p* values > 0.6). A similar analysis of accuracy rates across training days did not reveal a significant effect of day, *F*_4,72_ = 2.16, *p* = 0.08, but did reveal a significant main effect of training condition, *F*_4,72_ = 4.26, *p* = 0.022. Pairwise comparisons of the training conditions from the five days of behavioural training reveal that participants were on average 3.5% less accurate across days at identifying pairs of knots that were untrained, compared to the knots they were physically practicing tying (*p* = 0.015; Fig 3B).

Response time data collected during the pre- and post-training scanning sessions were submitted to a two (scanning session: pre-training and post-training) by three (training condition: tie, watch, untrained) repeated-measures ANOVA to assess for differences across scanning sessions and training conditions. No main effect of scan session was observed (*p* = 0.24), nor did a main effect of training condition emerge (*p* = 0.12). The interaction between scan session and training did not reach significance with a standard alpha value of 0.05 (*F*_2,34_ = 3.01, *p* = 0.06). As Fig 3C illustrates, however, this weak interaction between scan session and training experience appears to be at least partly driven by differences in reaction time in the pre-training scan, at the point when all knots were equally unfamiliar. This interaction is thus not considered further.

### Functional MRI results

#### Impact of physical or observational experience on object perception: Training experience x scan session interactions

**Fig 4:**
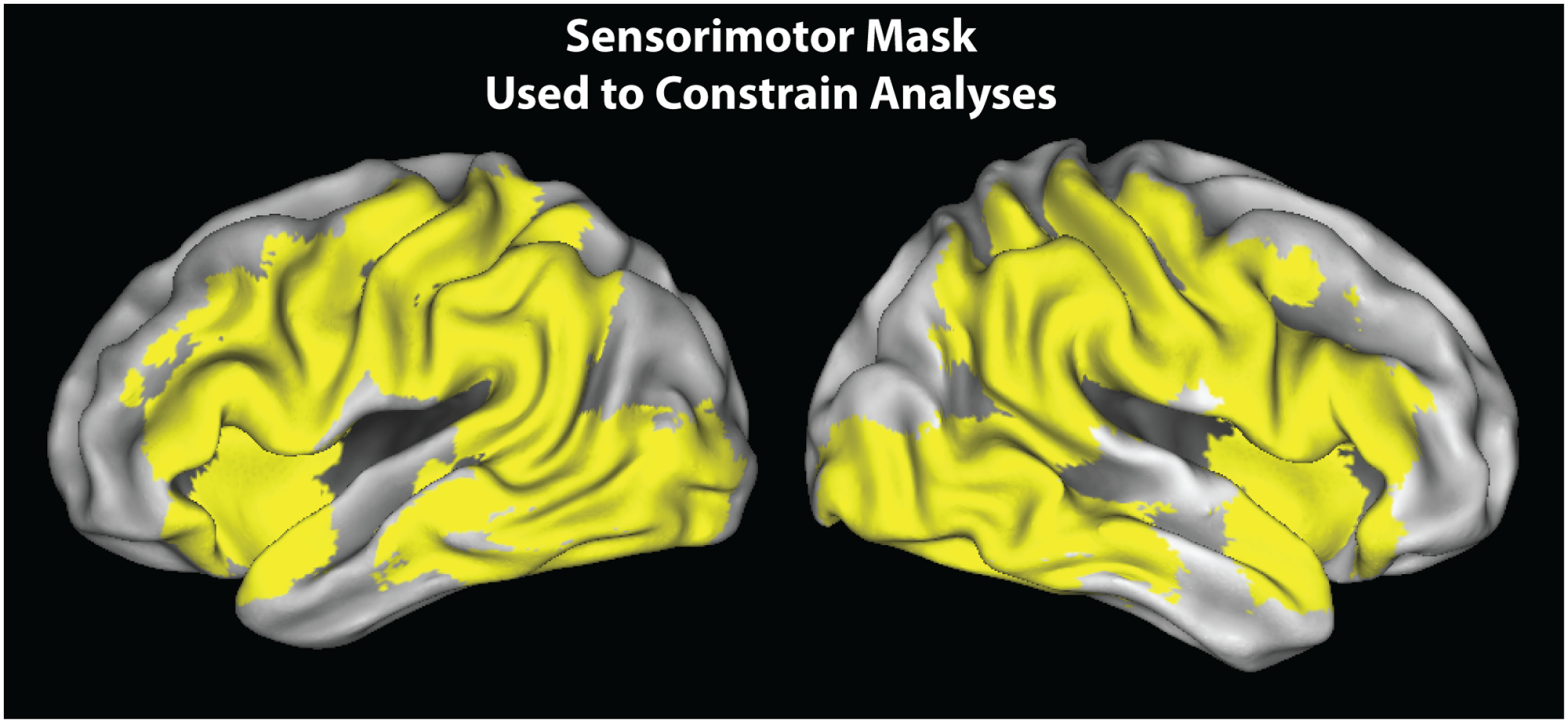
Sensorimotor mask used to constrain the search volume for evaluating the effects of the experimental design for all analyses. This anatomical mask was made by combining overlapping spheres 20 mm in diameter that covered bilateral IFG, IPL and posterior temporal brain regions, and has been used in previously published reports [39, 49].

**Fig 5:**
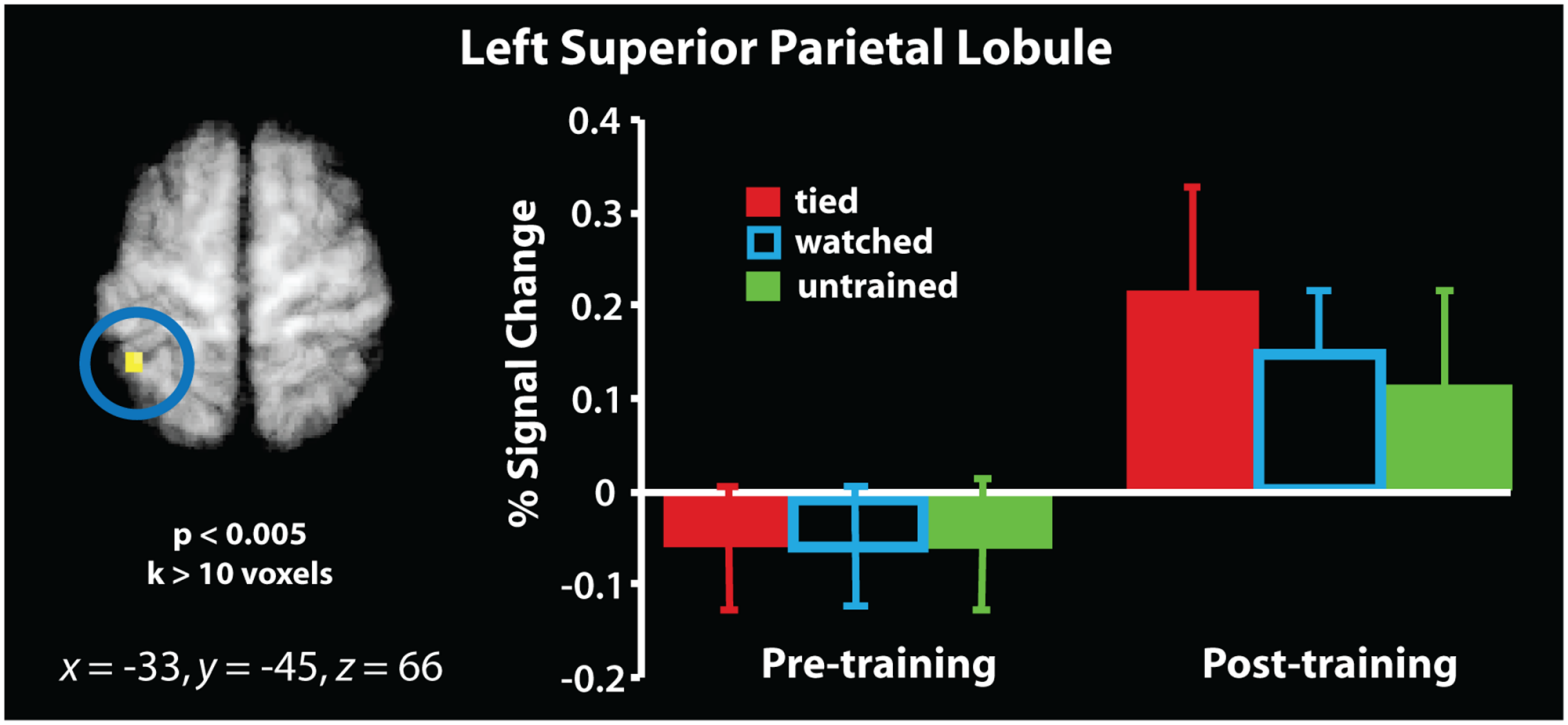
Interaction between physical training experience (learn to tie > untrained knots) and scanning session. The t-map has been evaluated a threshold *t* > 2.88, *p*_uncorr._ < 0.005, and is masked by the contrast illustrated in Fig 4 so that only those voxels that were part of the *a priori* established set of sensorimotor brain regions were interrogated. Parameter estimates were extracted from the resulting cluster from both pre-training and post-training scan sessions and are plotted to the right of the t-map for illustration purposes only. The outlined blue bars are included in the plot for completeness/illustration purposes only (i.e., the watched condition was not part of the contrast that is visualised on the brain image).

The mask within which all imaging analyses were evaluated is illustrated in Fig 4. With the first set of neuroimaging analyses, we examined the influence of training experience on object perception, we compared brain activity when participants saw trained (tied or watched) knots to brain activity when they saw untrained knots. These analyses were evaluated as interactions between scanning session and training experience, to determine whether any sensorimotor brain regions demonstrate a change in response between pre-training and post-training scans associated with a particular kind of training experience. The first interaction evaluated brain regions showing a greater response difference during the post-training scan session than the pre-training scan session when performing the perceptual discrimination task on knots from the ‘tied’ condition compared to those from the ‘untrained’ condition. This interaction demonstrated a stronger BOLD response within a region of the left superior parietal lobule spanning 20 voxels, centred on *x* = -33, *y* = -45, *z* = 66 with a *t*-value of 3.65 (Fig 5; see S1 Table for the results from a whole brain version of this interaction). We next ran a complementary interaction analysis to evaluate whether any brain regions demonstrated a scanning session by observational experience interaction for the ‘watched’ knots. No suprathreshold clusters emerged from this contrast.

#### Similarities between physical and observational experience on object perception

To investigate similarities between how physical and observational experience shape object perception, we next performed a conjunction analysis with custom-written MATLAB code to determine which voxels overlapped between these conditions after training. The most robust evidence for overlap between physical and visual experience would come from a conjunction analysis performed with two training experience (tying or watching) by scan session interaction contrasts (i.e., the two contrasts evaluated in the previous section). However, as presented in the previous section, we did not find evidence for any clusters demonstrating such an interaction for the knots that participants watched their partners train to tie. Moreover, prior imaging work demonstrates that such training by scan session interactions can be subtle, especially when the task involves a viewing static rather than dynamic displays of functional objects (c.f. [16, 28, 29]).

**Fig 6:**
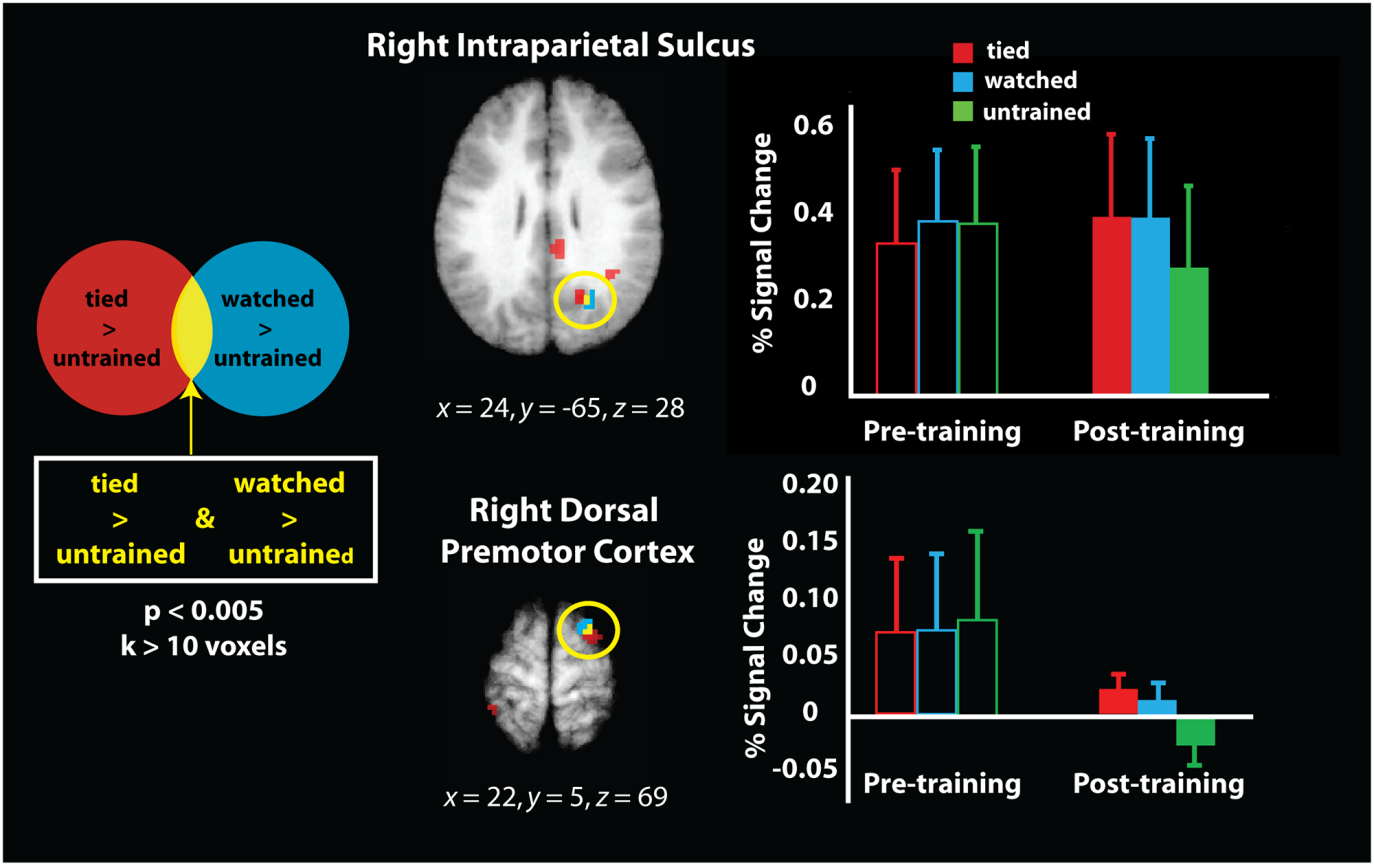
Direct comparison of the physical experience contrast (tied > untrained; in red) and the observational experience contrast (watched > untrained; in blue) from the post-training scan session, rendered on the same averaged brain image. This conjunction analysis highlights the two brain regions that emerged (overlap between red and blue regions, visualised in yellow) where both the tied > untrained and watched > untrained contrasts reached the significance threshold of *p*_uncorr._ < 0.005, masked by the sensorimotor brain regions illustrated in Fig 4. The plot illustrates the parameter estimates extracted from this region of the right IPS and right PMd. As this contrast was evaluated on the post-training scan session data only, and because all the knot categories were equally untrained/unfamiliar during the pre-training scan session, the percent signal change values from this same region during the pre-training scan session are visualised in outlined bars on the left side of the plot for illustration/comparison purposes only (i.e., these values were not part of the imaging contrast visualised to the left).

Therefore, to evaluate whether any brain areas demonstrated overlap between tied and watched knots only after the training intervention, we performed an exploratory conjunction analysis with the two contrasts (‘tied’ > ‘untrained’) and (‘watched’ > ‘untrained’) from the post-training scan session only. The brain regions that emerged from these exploratory individual contrasts from the post-training scan session are presented in Table 1. This conjunction analysis revealed that these two contrasts overlapped by 8 voxels (216 mm^3^) within the right intraparietal sulcus and right dorsal premotor cortex (Fig 6). Parameter estimates were extracted from these regions of overlap from each of the three training conditions from both the pre- and post-training scan sessions, as well as the scrambled knots visual control condition, and are illustrated in Fig 6. The pre-training parameter estimates are presented for illustration purposes only, to demonstrate both brain regions did not differentiate between the three training conditions pre-training. This was confirmed with two repeated-measures ANOVA on the week 1 parameter estimates, demonstrating no differences between the to-be-tied, to-be-watched, and to-remain-untrained conditions in the right IPS, *F*_2,36_ = 0.213, *p* = 0.809, or right PMd, *F*_2,36_ = 0.081, *p* = 0.923 (naturally, the parameter estimates from the ‘tied’ and ‘watched’ conditions in the post-training scan significantly differ from the parameter estimates from the ‘untrained’ condition, as this is the contrast the conjunction analysis was built from; thus, to avoid redundancy/double-dipping, these statistics are not reported). To test whether the magnitude of response within both regions reliably discriminated between pairs of tied or watched knots after training, we ran a paired samples t-test on the parameter estimates from these two conditions, for both regions. No significant difference emerged between the parameter estimates from the tied and watched conditions within right IPS, t(18) = -0.108, p = 0.916, or right PMd, t(18) = 0.443, p = 0.663. These results suggest that certain portions of parietal and premotor cortices show a similar response magnitude when viewing pictures of knots that had been associated with physical or visual experience after a week of training, and this response is stronger than when viewing the untrained knots. Further visualisation of these results can be found in S1 Fig, which illustrates the magnitude of individual participant’s percent signal change in these two brain regions across training conditions.

**Table 1:** Regions associated with discriminating pairs of knots with associated tying experience (compared to untrained knots) and pairs of knots with associated observational experience (compared to untrained knots).

Region	BA	MNI Coordinates			*t*-value	Cluster Size
		x	y	z		
*(a) Tying Experience > Untrained*						
R posterior superior frontal gyrus/PMd	6	27	-3	57	4.99	102
L calcarine gyrus	31	-21	-66	6	4.83	96
R precuneus	7	21	-63	27	4.49	31
L lingual gyrus	19	-15	-75	33	4.42	98
R inferior temporal gyrus	37	48	-42	-15	4.30	18
R angular gyrus/IPL	40	39	-66	45	4.16	115
L superior parietal lobule	7	-39	-48	66	3.81	62
R posterior superior frontal gyrus/PMd	6	24	3	72	3.70	14
L cerebellum lobule VIIIa		-33	-81	-30	3.69	18
L posterior middle frontal gyrus/pre-SMA	6	-3	15	57	3.66	12
L precentral gyrus	4	-30	-18	66	3.61	10
L middle temporal gyrus	37	-39	-69	12	3.54	37
R posterior cingulate cortex	23	9	-39	24	3.51	35
L inferior parietal lobule		-33	-63	45	3.43	11
*(b) Observational Experience > Untrained*						
R superior frontal gyrus/PMd	6	21	6	72	4.09	12
L inferior frontal gyrus	45	-48	36	12	3.73	15
R posterior intraparietal sulcus	7/19	24	-63	30	3.65	17

Results from the Tying Experience > Untrained (a), and Observational Experience > Untrained (b), masked by the sensorimotor region mask (Fig 4). Significance at all sites for each contrast was tested by a one-sample *t* test on β values averaged over each voxel in the cluster, with a significance threshold of p < 0.005 and k = 10 voxels. Coordinates are from the MNI template and use the same orientation and origin as found in the Talairach and Tournoux (1988) atlas. Contrasts from sections a and b were used to evaluate the conjunction analysis illustrated in Fig 6. Abbreviations for brain regions: PMd, dorsal premotor cortex; IPL, inferior parietal lobule; SPL, superior parietal lobule; pre-SMA, pre-supplementary motor area.

#### Differences between physical and observational experience on object perception

The final set of imaging analyses compared patterns of activity when discriminating knots from the ‘tied’ compared to the ‘watched’ conditions, as training experience by scanning session interactions. To focus on post-training effects from these interactions, the tied > watched by scan session interaction was masked by the main effect of tied > watched from the post-training scan session, and the watched > tied by scan session interaction was masked by the main effect of watched > tied from the post-training scan session. No clusters emerged from either of these training experience x scan session interactions. To further explore these data, we ran several additional post-hoc analyses to determine whether differences between tying and watching experience might emerge from contrasts that compare tying and watching experience to untrained knots or to the scrambled baseline condition. None of these contrasts ((tied > untrained) > (watched > untrained); (tied > baseline) > (watched > baseline); (watched > untrained) > (tied > untrained); (watched > baseline) > (tied > baseline)) yielded significant clusters, nor did inverse versions of these contrasts designed to test whether greater suppression in particular brain areas might reveal the harder task (i.e., (untrained > tied) > (untrained > watched); (untrained > watched) > (untrained > tied)).

## Discussion

The aim of the present study was to evaluate how observational and physical experience with previously-unfamiliar objects shapes neural activity when viewing those objects. Using a paradigm similar to that used in our previous work [16], we controlled the amount and type of experience participants gained while learning to tie different knots, all of which were equally unfamiliar prior to the start of the study. Such an approach enables precise quantification of the impact of different kinds of sensorimotor experience on object perception. When considering the impact of physical experience, imaging analyses revealed that five consecutive days of knot tying practice shaped the response within the left superior parietal lobule. After training, this region responded more robustly when discriminating between two knots that had been physically practiced compared to two knots that were untrained. A conjunction analysis examining overlap between knots from the ‘tied’ > ‘untrained’ and ‘watched’ > ‘untrained’ contrasts from the post-training scan session demonstrated that both physical and observational experience with knot tying shapes responses within a region of the right intraparietal sulcus and the right dorsal premotor cortex. Finally, direct contrasts between physical and observational experience did not yield evidence that any sensorimotor brain regions preferentially responded to discriminating knots from either training category after training compared to before training. Taken together, this pattern of findings, though preliminary in nature, suggests that both practical and visual experience with previously-unfamiliar objects shapes how such objects are perceived in an experience-dependant manner. In the following, we consider this pattern of findings in more detail after presenting some important caveats.

### A caution concerning interpretation of fMRI results at uncorrected thresholds

As reported in previous sections, the imaging results in the present study did not survive correction for multiple comparisons at the p < 0.05 level, with either familywise error (FWE) or false discovery rate (FDR) correction. A growing concern in the functional neuroimaging literature relates to the increased likelihood of false positive results when many statistical tests are performed over thousands of voxels simultaneously [51, 55, 56]. We acknowledge that the present imaging findings could indeed reflect a Type I error, and thus urge readers to interpret the findings with care.

Keeping this important caution in mind, we believe four factors justify publishing these results in the present form. First, it is important that marginal or ambiguous results are not relegated to a file drawer, where other researchers cannot access them (or include them in meta-analyses, for example, [57]). Second, the present study was designed in a hypothesis-driven fashion, carefully implemented, and finds weak effects within the predicted brain networks. If pre-registration had been common when the data were collected, we would have pre-registered the design and analyses as reported here. Third, learning studies can report small effects due to both signal increases and decreases with learning [58–62]. Relatedly, the use of static images of objects to assess visuomotor learning typically yields more subtle engagement of sensorimotor regions compared to tasks that involve viewing dynamic action displays (c.f., [16, 28, 40]). Finally, as only a handful of studies in this domain use training interventions with pre- and post-training fMRI measures, it is still relatively rare to have these kinds of training data to inform the field. We feel it is most honest to present our results as they are, within a framework where this pattern of fMRI effects was expected, and to encourage further investigation of this area. We welcome other laboratories to join us in attempting to replicate and explore these findings further.

### Impact of physical experience on object perception

Following the one-week training intervention, the tying experience by scan session interaction yielded a cluster of activity within left superior parietal lobule that was sensitive to the physical training manipulation when comparing pre- and post-training neural representations. This finding is consistent with what Weisberg et al. [28], Cross et al. [16], and Bellebaum et al. [24] report in similar physical experience by scanning session interactions with their novel object training manipulations. Such a finding not only fits well with the suggestion that the parietal cortex provides a pragmatic description of objects associated with actions [63, 64], but also that such action-related descriptions can be built *de novo* with just a few hours of practice across several days.

An extensive prior literature provides support for the notion that viewing static representations of objects that are strongly associated with particular actions (such as tools) engages the same sensorimotor brain regions activated when we use these objects [11, 14, 19, 65–67]. What is notable about the present findings is that we still find tentative evidence for engagement of one of these sensorimotor regions even when all objects hold the same functional value (i.e., all knots in our study were simply “knots” and had no functional value or association beyond that). As such, it appears that the manual experience participants had with each knot shaped its representation independently of any functional value. In other words, participants were not learning knots for specific rock climbing or sailing purposes, but were instead simply learning how to transform a piece of rope into a number of different three-dimensional shapes.

This pattern of findings corroborates and complements evidence reported from a recent study investigating how manual experience shapes object representations [68]. In this study, participants were required to view a series of objects and decide whether they were abstract or concrete objects while performing a concurrent manual task (continuously performing a series of patty-cake gestures). The authors found that participants experienced more task interference when viewing objects with which they had a great deal of manual experience (such as pencils, steering wheels, bananas and grapes) compared to objects with which they had little to no manual experience (including skunks, fences, garbage trucks and security cameras). Moreover, the self-reported amount of manual experience participants had with individual objects was directly related to how much interference they experienced. Recent work with left hemisphere stroke patients with apraxia further suggests that more superior and posterior portions of the left parietal cortex are associated with hand-object interactions more than with object function [69]. When these findings are considered with the present findings, a case emerges suggesting that manual experience (and not necessarily object function, *per se*) critically shapes object representations.

### Impact of observational experience on object perception

While findings from the physical practice condition add tentative support the generally agreed upon notion that physically interacting with objects shapes how these objects are perceived [70], an unresolved question concerns how observing *somebody else* interact with an unfamiliar object shapes perception of that object. According to the direct matching hypothesis [35, 36], physical experience with an action might not (always) be necessary for new action learning. The present study contributes to understanding how observational experience influences action representations in two ways. First, we show subtle evidence for engagement of sensorimotor cortices when participants view objects they observed their partner learn to create (c.f. [29]). In addition, we report preliminary evidence of responses with similar magnitudes within right IPS and PMd when participants discriminated knots from the ‘tied’ or ‘watched’ conditions, compared to the ‘untrained’ knots (see Fig 6 bar plots).

Keeping in mind the exploratory nature of these findings, the fact that we find some evidence to suggest observational experience plays a role in shaping object representations is perhaps more notable due to the fact that participants were not *explicitly* asked to learn to tie those knots they watched their partner learn, and yet they can tie their partners’ knots significantly better than untrained knots after training (contrast this to weaker behavioural effects of observational learning reported in, e.g., [39]). We suggest that participants’ observational learning in the present study was likely bolstered by watching a live model (their partner) transition from novice to expert status as a knot tyer, as prior research suggests observing both experts and novices at a given motor task leads to improved observational learning of that same task [71, 72]. Future investigations into how observational experience shapes object representations could vary how much information participants know about the task and later performance expectations, as evidence suggests that more information about the task or future performance evaluation benefits observational learning [73, 74].

One final point worth noting concerns the overlapping engagement found within PMd for watched and tied knots. Engagement of this region based on visual and visuomotor experience is consistent with prior reports of PMd involvement in sequential visuomotor association training [41, 75–77]. As such, one likely interpretation for the present pattern of findings is that engagement of this region reflects learned associations between sequential motor behaviour and object shapes. It would be instructive for future work to explore varying the functional utility of novel objects to disentangle the role of motor sequence learning and functional object knowledge when viewing previously novel objects.

### Limitations and future directions

In addition to the caution we urge in interpreting imaging results at non-corrected thresholds, several additional issues require consideration and also present rich opportunities for follow-up research. One of these issues concerns the specificity of our training manipulation. Could the effects we report arise from general changes in familiarity, recognition, or task parameters (e.g., explicit vs. implicit learning) and thus be non-specific to the physical or observational experience? To precisely control familiarity, it would be possible to include a training condition where participants view knots without tying them or observing them being tied. We included such a condition in our previous study (Cross et al., 2012) and found that motor experience engages bilateral IPS beyond perceptual experience alone (see also the visual exploration conditions of Bellebaum et al. [24] and Rüther et al. [29]). This suggests that parietal activation is unlikely to be driven solely by visual familiarity, but further study is still needed to directly test this in the context of observational learning. Moreover, as the present study contrasted explicit physical learning with implicit visual learning, another important direction for future research will be to more carefully assess the intention to learn. For example, a follow-up study exploring explicit physical vs. explicit observational learning could shed more light on the role of a learner’s intention in shaping practical object representations (c.f., [78]).

Another valuable future direction involves making use of fMRI approaches that enable more sensitive investigation of the influence of experience on (object) perception. As mentioned previously, prior studies on sensorimotor learning document both increases and decreases in frontoparietal brain regions following training, with increases argued to reflect increased recruitment of cortical tissue and decreases suggestive of more efficient neural function [58, 61]. However, because conventional fMRI analyses average activity across voxels in a particular brain region to explore how learning changes the magnitude of a given brain region’s response, they are insensitive to a richness of information that is represented by more subtle patterns of activity distributed across voxels [79]. As such, the coarseness of conventional fMRI analyses limits understanding of neural function by disregarding the multidimensional nature of representational space. It would thus be valuable for follow-up work to use multi-voxel pattern analysis (MVPA) to identity the extent to which patterns of activity across voxels within a brain region are distinct between different training conditions, independent of average activity [79, 80]. Such an approach should be particularly well-suited for examining the impact of physical and visual learning on sensorimotor cortical activity (e.g., [81]).

One final important consideration for the current and future studies concerns our treatment of knots as unfamiliar functional objects that are in some meaningful way akin to tools. Most research that has examined neural representations of functional objects has focused on canonical, rigid tools that hold their shape while being manipulated in a repetitive manner, often while interacting with another object, such as hammers (which are most often used to pound nails), axes (used to chop wood), cutlery (used to cut food), scissors (used to cut paper), etc. [13, 14, 18, 21, 65, 82]. Studies of novel object function have typically used objects made of moveable rigid parts designed to be used to interact with other objects [22, 24, 27, 29]. Importantly, most theoretical accounts of functional object perception also focus on these kinds of tools [64, 83, 84]. Therefore, it is important to consider whether knots are similar to the rigid tools studied by other groups. We cannot answer this question without directly comparing learning about rigid and non-rigid objects, but considering that non-rigid objects may have more complex dynamics and more potential to do a number of different things compared to rigid objects, we consider them to be a useful research stimulus, and future studies may wish to further explore object perception using other non-rigid materials/objects, such as origami or modelling clay.

### Conclusion

When considered together, the findings from the present study add preliminary support to the idea that physical experience, and in a subtler manner, purely observational experience as well, impact the recruitment of sensorimotor brain regions during object perception. The impact of physical experience on sensorimotor engagement yielded a training experience by scan session interaction within left SPL. In contrast, the findings from the observational experience contrasts were subtler, only evident during an exploratory contrast run on post-training object perception. This pattern of findings suggests that prior experience with an object, whether visuomotor or visual in nature, shapes subsequent perception of that object. Through use of a perceptual discrimination task where participants were not required to explicitly access their previous experience, we found that participants’ observational or physical training experience became an integral feature of these objects that was engaged during perception. The present findings complement developmental work showing that neonates acquire knowledge about novel objects from observing others interacting with them [85–87], and highlight the utility of using training paradigms with previously unfamiliar objects to probe the emergence of action-related object representations in the adult brain. Naturally, we acknowledge that it is not possible to directly compare the short-term learning effects studied in the present study with how real-world experience shapes object perception in development. We do, however, agree with several authors before us [16, 22, 24, 27, 29] that such learning paradigms offer a powerful tool for studying the emergence of object representations at brain and behavioural levels.

## Supporting information

S1 Fig Parameter estimate plots for the 19 individual participants composing the participant sample illustrated in the conjunction analysis in Fig 6 of the main text. As can be seen from these plots, there is a large degree of variability across participants, with 14/19 showing a tied>untrained main effect and 15/19 showing a clear watched > untrained main effect in right IPS (top plots), and within the right PMd ROI, we see 14/19 showing the main effect of greater activity for the tied > untrained knots, and 11/19 showing a main effect for watched > untrained knots. Looking more specifically at individual participants whose brain activity reflects the actual conjunction analysis illustrated in Fig 6 of the main text, it would appear that 13/19 demonstrate a pattern of more robust engagement within right IPS when viewing knots that had been tied compared to untrained AND knots that had been watched compared to untrained, while 9/19 demonstrate the same pattern within right PMd.
(DOCX)

S1 Table Whole brain analysis of the interaction between physical training experience (learn to tie > untrained knots) and scanning session. This table lists the brain regions that emerge from a whole-brain version of the (tying) training experience by scan session interaction, illustrated in Fig 5 of the main text.
(DOCX)
