# Supplementary material for: Learning to tie the knot: The acquisition of functional object representations by physical and observational experience
Source: PLoS One. 2017 Oct 12;12(10):e0185044. doi: 10.1371/journal.pone.0185044 (PMC5638238; doi:10.1371/journal.pone.0185044)

**S1 Fig. Parameter estimate plots for the 19 individual participants composing the participant sample illustrated in the conjunction analysis in Figure 6 of the main text.** As can be seen from these plots, there is a large degree of variability across participants, with 14/19 showing a tied>untrained main effect and 15/19 showing a clear watched > untrained main effect in right IPS (top plots), and within the right PMd ROI, we see 14/19 showing the main effect of greater activity for the tied > untrained knots, and 11/19 showing a main effect for watched > untrained knots. Looking more specifically at individual participants whose brain activity reflects the actual conjunction analysis illustrated in Fig 6 of the main text, it would appear that 13/19 demonstrate a pattern of more robust engagement within right IPS when viewing knots that had been tied compared to untrained AND knots that had been watched compared to untrained, while 9/19 demonstrate the same pattern within right PMd.


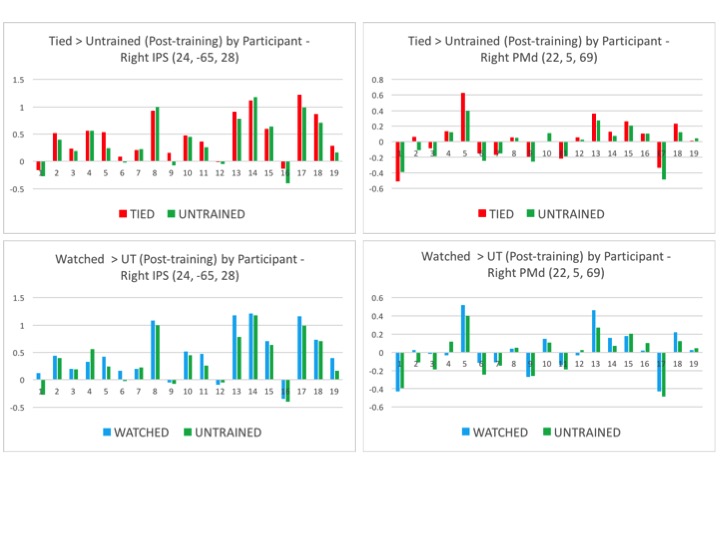

Supplement: S1 Fig — As can be seen from these plots, there is a large degree of variability across participants, with 14/19 showing a tied>untrained main effect and 15/19 showing a clear watched > untrained main effect in right IPS (top plots), and within the right PMd ROI, we see 14/19 showing the main effect of greater activity for the tied > untrained knots, and 11/19 showing a main effect for watched > untrained knots. Looking more specifically at individual participants whose brain activity reflects the actual conjunction analysis illustrated in Fig 6 of the main text, it would appear that 13/19 demonstrate a pattern of more robust engagement within right IPS when viewing knots that had been tied compared to untrained AND knots that had been watched compared to untrained, while 9/19 demonstrate the same pattern within right PMd. (DOCX) [file pone.0185044.s001.docx]
