# Supplementary material for: Learning to tie the knot: The acquisition of functional object representations by physical and observational experience
Source: PLoS One. 2017 Oct 12;12(10):e0185044. doi: 10.1371/journal.pone.0185044 (PMC5638238; doi:10.1371/journal.pone.0185044)
Supplement: S1 Table — This table lists the brain regions that emerge from a whole-brain version of the (tying) training experience by scan session interaction, illustrated in Fig 5 of the main text. (DOCX) [file pone.0185044.s002.docx]

**S1 Table. Whole brain analysis of the interaction between physical training experience (learn to tie > untrained knots) and scanning session.** This table lists the brain regions that emerge from a whole-brain version of the (tying) training experience by scan session interaction, illustrated in Fig 5 of the main text.

| Region | BA | MNI Coordinates | | | *t*-value | Cluster Size |  |  |  |  |
| --- | --- | --- | --- | --- | --- | --- | --- | --- | --- | --- |
|  |  | x | y | z |  |  |  |  |  |  |
| *(a) Post-Training Scan (Tying Experience > Untrained) > Pre-Training Scan (To-Be Tied > To-Remain Untrained)* | | | | | | | |  |  |  |
| Midline cuneus/V2 | 19 | 0 | -99 | 18 | 4.94 | 48 |  |  |  |  |
| R fusiform gyrus | 37 | 30 | -63 | -9 | 4.60 | 176 |  |  |  |  |
| L para-angular gyrus | 39 | -39 | -48 | 12 | 4.38 | 28 |  |  |  |  |
| L lingual gyrus | 18 | -24 | -72 | -3 | 4.13 | 36 |  |  |  |  |
| L superior parietal lobule | 7 | -33 | -45 | 63 | 3.65 | 28 |  |  |  |  |
| L cerebellum |  | -33 | -87 | -33 | 3.53 | 13 |  |  |  |  |
